# Cerebral Salt-Wasting Syndrome Caused by Minor Head Injury

**DOI:** 10.1155/2017/8692017

**Published:** 2017-01-17

**Authors:** Toshiki Fukuoka, Yuko Tsurumi, Arihito Tsurumi

**Affiliations:** ^1^Department of Neurosurgery, Nagoya University Graduate School of Medicine, Nagoya, Japan; ^2^Department of Neurosurgery, Tsurumi Neurosurgery, Koga, Ibaraki, Japan

## Abstract

A 34-year-old woman was admitted to hospital after sustaining a head injury in a motor vehicle accident (day 1). No signs of neurological deficit, skull fracture, brain contusion, or intracranial bleeding were evident. She was discharged without symptoms on day 4. However, headache and nausea worsened on day 8, at which time serum sodium level was noted to be 121 mEq/L. Treatment with sodium chloride was initiated, but serum sodium decreased to 116 mEq/L on day 9. Body weight decreased in proportion to the decrease in serum sodium. Cerebral salt-wasting syndrome was diagnosed. This case represents the first illustration of severe hyponatremia related to cerebral salt-wasting syndrome caused by a minor head injury.

## 1. Introduction

Hyponatremia resulting from cerebral salt-wasting syndrome (CSWS) can occur after severe brain injury, severe cerebrovascular disease, or surgery [1–9]. Hyponatremia can result in brain edema and secondary nausea, headache, altered consciousness, and sometimes death. Close monitoring of serum Na levels and immediate correction of electrolyte abnormalities are therefore necessary after severe brain damage. If left untreated without correct diagnosis, severe hyponatremia may result in seizures and worsening cerebral edema [10].

However, no previous reports have described hyponatremia of CSWS occurring after minor head injury in the absence of intracranial bleeding, skull fracture, or brain contusion. This report describes the case of a patient with minor head injury who developed severe hyponatremia due to CSWS.

## 2. Case Report

A 34-year-old woman with no significant past medical history sustained an injury to the right forehead in a motor vehicle accident (day 1). She was not taking any regular medications. Physical examination revealed no traumatic wounds other than a thin subcutaneous hematoma on the right forehead. She presented with headache and nausea, and Glasgow coma scale score was 14 (E3, V5, M6), but no obvious focal neurological signs were present, including amnesia. Furthermore, computed tomography (CT) of the head revealed no skull fracture, intracranial hemorrhage, or brain contusion. Complete blood cell (CBC) count and serum biochemistry revealed no abnormalities, and serum Na concentration was normal (141 mEq/L). She was hospitalized for observation under a diagnosis of brain concussion. By day 2, Glasgow coma scale score had normalized and symptoms of headache and nausea had almost resolved. On day 3, serum Na was still within the normal range but had decreased to 135 mEq/L. CBC and serum biochemistry revealed no abnormalities, and the patient was discharged without symptoms on day 4.

After discharge from hospital, she began to feel severe and gradually worsening fatigue and nausea and finally presented to the emergency department on day 8. Head CT revealed no abnormal findings. Blood testing disclosed serum Na of 121 mEq/L and serum Cl of 90 mEq/L, while serum biochemistry showed no other abnormalities. Skin turgor was slightly diminished, suggesting decreased circulating plasma volume. She was therefore hospitalized for evaluation and management of hyponatremia.

Treatment was initiated via intravenous saline and oral administration of salt with frequent monitoring of serum Na levels. Body weight was measured daily to help distinguish between the presence of CSWS, syndrome of inappropriate secretion of antidiuretic hormone (SIADH), and other disorders. Blood testing on day 9 revealed serum Na of 116 mEq/L and serum osmolality of 251 mOsm/L (reference range: 275–285 mOsm/L). On day 10, blood testing showed serum Na of 125 mEq/L (urine Na: 65 mEq/L), serum osmolality of 251 mOsm/L (urine osmolality: 238 mOsm/L), a urine acid level of 1.5 mg/dL (reference range: 2.3–7.0 mg/dL), an atrial natriuretic peptide (ANP) level of 114 pg/mL (reference range: <43 pg/mL), a brain natriuretic peptide (BNP) level of 142 pg/L (reference range: 0–18 pg/L), an antidiuretic hormone (ADH) level of 0.79 pg/mL (reference range: 0.3–4.2 pg/mL), a plasma renin activity level of <2.0 pg/mL (reference range: 2.5–21.4 pg/mL), a thyroid-stimulating hormone (TSH) level of 2.734 *μ*g/mL (reference range: 0.350–4.940 *μ*g/mL), a free triiodothyronine level of 2.24 pg/mL (reference range: 1.71–3.71 pg/mL), a free thyroxine level of 1.02 ng/dL (reference range: 0.70–1.48 ng/dL), a morning fasting hydrocortisone level of 14.99 *μ*g/mL (reference range: 6–20 *μ*g/mL), and an adrenocorticotropic hormone level of 30.2 pg/mL (reference range: 7.4–55.7 pg/mL). In addition, body weight decreased as serum Na decreased.

**Figure 1:**
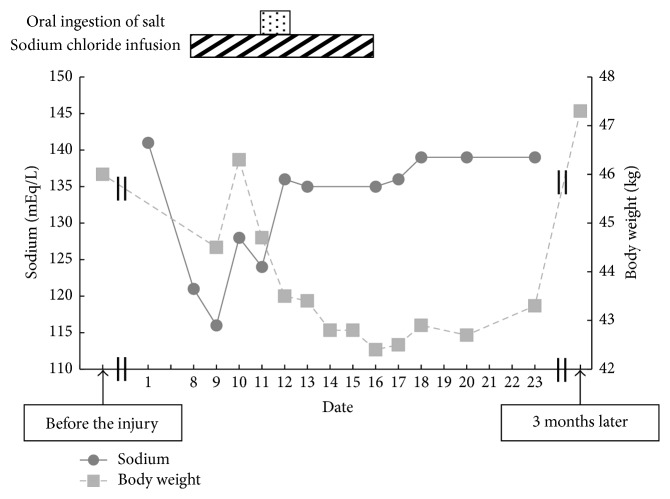
Clinical course of treatment and examinations.

These results, the diminished skin turgor, and the decrease in body weight indicated a diagnosis of CSWS and the absence of renal failure, thyroid dysfunction, and adrenal insufficiency. Blood testing showed serum Na of 129 mEq/L on day 11 (urine Na: 60 mEq/L) and serum Na of 136 mEq/L (urine Na: 55 mEq/L) on day 12. Serum Na subsequently remained within the normal range. On day 16, intravenous saline infusion was terminated. Fatigue and nausea resolved as serum Na concentrations increased. After day 20, body weight started to improve towards baseline. She was discharged on day 24 without any subsequent recurrence of hyponatremia. Serum BNP level on day 27 had completely normalized to 10 pg/L (reference range: 0–18 pg/L). The course of treatment is shown in Figure 1.

## 3. Discussion

CSWS is characterized by renal loss of sodium following intracranial disorders, resulting in hyponatremia and hypovolemia [11–13]. CSWS ordinarily occurs after severe brain injury, severe cerebrovascular disease, or surgery [1–9], and no previous reports have described CSWS after minor head injury. Intriguingly, lightning injury [14] and therapeutic barbiturate coma [15] may also cause CSWS. While many studies have described aspects of CSWS, the pathogenesis of renal salt wasting derived from cerebral disease is not fully understood. The most probable process involves disruption of neural inputs to the kidney and/or central production of a circulating natriuretic factor [6, 7, 11, 12, 16, 17]. In addition, some authors have indicated that ANP and BNP exert biologic effects that could lead to CSWS [3, 6, 7, 18–20]. The time from traumatic brain injury to development of CSWS can vary from 2 days to 2 months [2, 3, 6, 18, 21, 22]. Regarding the severity of CSWS, highly invasive surgery and the severity of findings on head CT are associated with the severity of CSWS [9, 21]. In other words, CSWS is unlikely to occur in the absence of severe brain damage.

Differentiating between CSWS and SIADH is critical for appropriate treatment of hyponatremia, because therapeutic strategies for the two syndromes differ markedly. When hyponatremia is treated inappropriately, the patient is at increased risk of delayed ischemic deficits and/or osmotic demyelination leading to disability and mortality [23]. The primary distinction between CSWS and SIADH is whether the circulating blood volume is decreased or increased [6, 12, 24, 25]. Since some CSWS cases do not present with characteristic physical findings, comprehensive judgment is frequently needed to reach a definitive diagnosis. In the present case, serum Na concentrations, osmolality, ANP, BNP, ADH, diminished skin turgor, decreasing body weight, and prolonged natriuresis despite hyponatremia were consistent with the diagnosis of CSWS rather than SIADH. As a matter of fact, serum Na levels and symptoms were greatly improved with substantial hydration and NaCl administration.

CSWS is generally caused by severe brain injury or severe cerebrovascular disease, and head CT and magnetic resonance imaging typically reveal abnormal findings. In the present case, head CT was notable for the absence of intracranial bleeding and brain contusion. This case shows that even minor head injuries can cause disruptions to the neural inputs to the kidney and/or central production of circulating natriuretic factors that eventually contribute to CSWS. Clinicians should be aware that even minor head injury may result in CSWS, hyponatremia, and secondary symptoms.
